# Distributed MPC of vehicle platoons with guaranteed consensus and string stability

**DOI:** 10.1038/s41598-023-36898-4

**Published:** 2023-06-27

**Authors:** Yangyang Feng, Shuyou Yu, Hao Chen, Yongfu Li, Shuming Shi, Jianhua Yu, Hong Chen

**Affiliations:** 1 Department of Control Science and Engineering, Jilin University, Changchun, 130022 China; 2 State Key Laboratory of Automotive Simulation and Control, Jilin University, Changchun, 130022 China; 3 College of Automation, Chongqing University of Posts and Telecommunications, Chongqing, 400065 China; 4 College of Transportation, Jilin University, Changchun, 130022 China; 5 Dongfeng Commercial Vehicle Technology Center, Dongfeng Motor Corporation, Wuhan, 442001 China; 6 College of Electronic and Information Engineering, Tongji University, Shanghai, 201804 China

**Keywords:** Electrical and electronic engineering, Mechanical engineering

## Abstract

Control of vehicle platoon can effectively reduce the traffic accidents caused by fatigue driving and misoperation, reduce air resistance by eliminating the inter-vehicle gap which will effectively reduce fuel consumption and exhaust emissions. A hierarchical control scheme for vehicle platoons is proposed in this paper. Considering safety, consistency, and passengers’ comfort, a synchronous distributed model predictive controller is designed as an upper-level controller, in which a constraint guaranteeing string stability is introduced into the involved local optimization problem so as to guarantee that the inter-vehicle gap error gradually attenuates as it propagates downstream. A terminal equality constraint is added to guarantee asymptotic consensus of vehicle platoons. By constructing the vehicle inverse longitudinal dynamics model, a lower-level control scheme with feedforward and feedback controllers is designed to adjust the throttle angle and brake pressure of vehicles. A PID is used as the feedback controller to eliminate the influence of unmodeled dynamics and uncertainties. Finally, the performance of longitudinal tracking with the proposed control scheme is validated by joint simulations with PreScan, CarSim, and Simulink.

## Introduction

Control of vehicle platoon has significant social and economic value for improving vehicle driving safety, energy-saving, and emission reduction. It can reduce the labor intensity of drivers, avoid traffic accidents caused by drivers’ misoperation or illegal operation^1^. Control of vehicle platoon can effectively reduce the inter-vehicle gap, so that the following vehicles will enter the wake region under the barrier of the leader vehicle. That is, vehicle platoon can reduce the air resistance of the vehicle at a high speed, and reduce fuel consumption and exhaust emissions^2^.

A hierarchical control strategies for vehicle platoon is proposed in^3,4^, in which the upper-level control method plans the motion and path, and the lower-level control method executes the commands of upper-level control method. In^5^, a hierarchical control structure for vehicle platoon is proposed, where the upper-level control method performs distributed control, and the lower-level control method adopts the feedback linearization technology to achieve drive and brake control. In^6^, a hierarchical control structure is adopted for vehicle platoons with actuator delays and non-ideal communication conditions. The upper-level distributed proportional controller guarantees string stability, and the lower-level adopts the inverse model-based feedforward control to regulate the driving and braking of vehicles. In^7^, the upper-level utilizes model predictive control (MPC), in which a multi-vehicle collision avoidance system is proposed to minimize the risk of collision. In^8^, considering the complexity of network-connected vehicle platoon, a simplified model of vehicles is utilized to design the upper-level controller to achieve string stability, reduce fuel consumption, and collision avoidance, etc. In the lower-level, an adaptive control strategy is implemented to regulate engine torque and switching gears. In^9^, an adaptive sliding mode control method is chosen as the lower-level controller so as to guarantee tracking performance in the case of disturbances. In^10^, a hierarchical personalized adaptive cruise controller is proposed, where the upper-level controller adopts MPC, and the lower-level controller adopts the combination of feedforward and feedback. Currently, the research on hierarchical control structure of vehicle platoons mainly focuses on exploring the upper-level strategies, such as the influence of vehicle platoon model, inter-vehicle gap strategy, communication topology, communication time delay, and string stability, etc^11^. However, the research on the lower-level control strategies of vehicle platoons is still relatively limited. The lower-level controllers are designed often utilizing simple models and inverse engine characteristic maps to “control” the vehicle throttle angle and brake pressure^12^. In this paper, a lower-level controller with the feedforward and feedback control strategies is proposed, where the feedforward controller is designed as well based on an inverse longitudinal dynamics model of vehicles, and the feedback controller eliminates the influence of model uncertainties and unmodeled dynamics by adopting a PID controller.

Model predictive control is widely applied to Advanced Driver-Assistance Systems since it can generally provide better performances than standard control methods^13^. Distributed model predictive control (DMPC) is a kind of model predictive control, which can predict the future control sequence as the future estimate of each vehicle to improve the control effect of vehicle platoons^14^. In-depth research on distributed mode predictive control has been conducted to design corresponding coordination strategies according to different performance requirements, which involves coupling constraints of the system, stability, security, and feasibility analysis, etc^15^. In general, DMPC converts the control problem into an optimization problem so as to obtain control actions accordingly. In^16^, a DMPC algorithm based on Nash optimality is proposed, which achieves better performance by exchanging information (communication) during the optimization process. In^17^, a distributed economic MPC strategy is constructed to minimize the fuel consumption of vehicle platoons. In^18^, a DMPC method is desigen for the vehicle platoon under unidirectional communication topologies. In^19^, a DMPC algorithm is adopted for the vehicle platoon under switching communication topologies. To ensure asymptotic stability, a terminal equality constraint is added, which enforces the terminal state of each vehicle to be equal to the average state of its neighbours^18–22^. Note that some investigations also use the terminal inequality constraint to analyze the asymptotic stability of DMPC^23–26^. Though the terminal inequality constraint is easier to implement numerically compared to the terminal equality constraint, there are many highly efficient methods for solving optimization problem with terminal equality constraints^27^.

String stability of vehicle platoons must also be considered^28^. Recently, research on string stability of vehicle platoons is mainly focused on the frequency domain^29,30^. However, DMPC algorithm of vehicle platoons is difficult to guarantee the constraint satisfaction in the frequency domain; moreover, converting the frequency domain analysis of string stability into the time domain is difficult in general^31^. String stability of a platoon of vehicles with nonlinear dynamics by using the DMPC method is first proposed in^27^, which transforms string stability requirement into an inequality constraint. And sufficient conditions are given to ensure string stability for both leader−follower communication topology and predecessor−follower communication topology. In^32^, a new DMPC scheme is designed for the heterogeneous vehicle platoon with input and state constraints to ensure the closed-loop stability and $\gamma$-gain string stability (a new string stability concept). A distributed economic MPC algorithm is proposed in^33^ to ensure asymptotic stability, and to achieve $\gamma$-gain string stability simultaneously.

This paper proposes a synchronous DMPC algorithm with guaranteed string stability as the upper-level controller for vehicle platoons. Each vehicle constructs a local optimization problem based on communication topology, and solves its local optimization problem synchronously to obtain a feasible solution. Combining with the proposed lower-level control strategy, the string stability and consensus of vehicle platoons are verified by the joint simulation with PreScan, CarSim and Simulink. The main highlights of this paper are listed below:

1. In this paper, a synchronous DMPC algorithm of a vehicle platoon is proposed, and the string stability with predecessor-leader following (PLF) communication topology is investigated. By adding an inequality constraint to the optimization problem, the string stability of vehicle platoons is guaranteed. In addition, a terminal equality constraint is added to guarantee the asymptotic consensus of vehicle platoons.
2. Considering the real scenarios of the vehicle platoon, the desired control input determined by the upper-level DMPC cannot be directly implemented on the real vehicle. Therefore, a feedforward and feedback control strategy is designed. The feedforward controller is based on the vehicle inverse longitudinal dynamics model, which transforms the desired control input into throttle angle and brake pressure, and the feedback controller is designed to eliminate the influence of model uncertainties and unmodeled dynamics.

The remainder of the paper is structured below. Section II sets up the problem, including communication topology, vehicle dynamics, vehicle platoon modeling, control objective. Section III presents the hierarchical control structure for the vehicle platoon, which includes an upper-level distributed model predictive control, and a lower-level feedforward and feedback control strategies. Section IV is the joint simulation with PreScan, CarSim and Simulink. Section V ends the paper with conclusions.

Notation: Denote ${N_{\left[ {{k_1},\,{k_2}} \right] }} = \left\{ {{k_1},\,{k_1} + 1,\,\cdots ,\;{k_2}} \right\}$, both ${k_1}$ and ${k_2}$ are integer, ${k_2} > {k_1}$. Define ${\left\| {\vartheta (t)} \right\| _2}$ as the 2-norm of the function ${\vartheta (t)}$, i.e., $\mathop {\mathrm{{lim}}}\limits _{t \rightarrow \infty } \,\vartheta (t) = 0$.

## Vehicle platoon and problem setup

This section first introduces the PLF communication topology, then the vehicle and vehicle platoon models. Since the focus of the paper is the longitudinal control of a platoon, the vehicle dynamics model is simplified below:

1. Only the longitudinal motion of vehicles is studied, i.e., the lateral and vertical motion of vehicles are ignored.
2. Neither the slippery roads nor vehicle tires slipping are taken into account.

### Communication topology

The vehicle in the platoon needs to know itself, and its neighbouring vehicles’ information. The vehicle obtains its status information such as position, velocity, etc., through onboard sensors or state estimation. Through V2V communication, a connection is established with neighbouring vehicles in the platoon.

**Figure 1:**
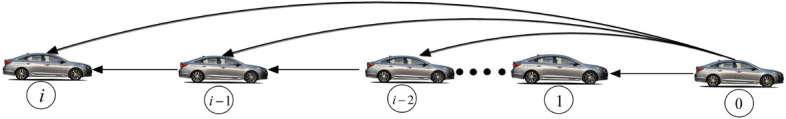
The structure of the PLF communication topology.

A vehicle platoon with one leader vehicle and *M* following vehicles, which is driving in a straight line. The PLF communication topology is employed, a vehicle platoon under the PLF communication topology is illustrated in Fig. 1.

### Vehicle dynamics

The *i*th vehicle’s longitudinal dynamics is formulated by a $3^{rd}$ model^34^

1 $$\begin{aligned} \left\{ {\begin{array}{*{20}{l}} {{{\dot{q}}_i} = {v_i}}\\ {{{\dot{v}}_i} = \frac{1}{{{m_i}}}\left( {\frac{{{\eta _{T,i}}}}{{{r_{eff,i}}}}{T_i} -\frac{1}{2}{C_{d,i}}{{\bar{A}}_i}\mu _i {v_i}^2 - {m_i}g{f_i}} \right) }\\ {{{\dot{T}}_i} = - {\sigma _i}^{ - 1}{T_i} + {\sigma _i}^{ - 1}{T_{des,i}}} \end{array}} \right. \end{aligned}$$

where $i \in {N_{\left[ {1,\,M} \right] }}$, ${q_i}$ is the position of the $ith$ vehicle; ${q_i}$ is the velocity of the $ith$ vehicle; ${{T_i}}$ and ${{T_{des,i}}}$ represent the actual and desired drive/braking torque, respectively; ${C_{d,i}}$ is the aerodynamic drag coefficient; $\mu _i$ is the ambient air density; ${m_i}$ is the vehicle mass; ${{\bar{A}}_i}$ is the frontal area; ${{{\sigma } _i}}$ is the time constants of longitudinal dynamics; ${{\eta _{T,i}}}$ is the mechanical efficiency of driveline; ${{r_{eff,i}}}$ is the effective rolling radius; $f_{i}$ is the rolling resistance coefficient; *g* is the gravity acceleration.

Assume that the aforementioned parameters are known, then the nonlinear control law^28,35^ is designed accordingly

2 $$\begin{aligned} {T_{des,i}} = \frac{{{r_{eff,i}}}}{{{\eta _{T,i}}}}\Big ( {{m_i}{u_i} + {m_i}g{f_i} +\frac{1}{2}{C_{d,i}}{{\bar{A}}_i}\mu _i {v_i}\left( {2{\sigma _i}{a_i} + {v_i}} \right) } \Big ) \end{aligned}$$

Combining (1) and (2), the linear vehicle model can be obtained

3 $$\begin{aligned} \left\{ {\begin{array}{l} {{\dot{q}}_i} = {v_i}\\ {{\dot{v}}_i} = {a_i}\\ {{\dot{a}}_i} = - {{\sigma }_i}^{ - 1}{a_i} + {{\sigma }_i}^{ - 1}{a_{des,i}} \end{array}} \right. \end{aligned}$$

where $${a_i}$$ is the acceleration; $$ {{a_{des,i}}}$$ the desired acceleration, i.e., the control input.

For simplicity, the following assumptions are made in the paper.

#### Assumption 1

For any vehicle $i,\,i \in {N_{\left[ {1,\,M} \right] }}$, its position $q_i$, velocity $v_i$, and acceleration $a_i$ can be measured instantaneously.

#### Assumption 2

Only the longitudinal motion of the vehicle is studied, i.e., the lateral and vertical motion of the vehicle are ignored.

#### Assumption 3

Each vehicle shares a synchronized clock, i.e., the onboard controllers are synchronized.

#### Remark 1

Since the knowledge of states and parameters plays a crucial role in the controller design of vehicles^36^, state estimation and sensor fusion of vehicles in the platoon will be our future research direction.

### Vehicle platoon modeling

Suppose that the leader vehicle is uncontrolled, cf., its position and velocity are given as $\left( {{q_0}(t),{v_0}(t)} \right)$. For following vehicle *i*, $i \in 1,\,2,\, \cdots ,\,M,$ describe its position and velocity as $\left( {q_i}(t),{v_i}(t)\right)$, and define the reference position and velocity are

$$\begin{aligned} \big ( {{q_0} (t) - i {q_ {{{des}}}}, {v_0} (t) }\big ) \end{aligned}$$

where $${q_ {{{des}}}} $$ is the desired inter-vehicle gap.

In this paper, the constant distance policy^37^ is adopted

4 $$\begin{aligned} {q_{des}} = q_0 \end{aligned}$$

with $$q_0>0$$.

According to the current position and reference position of the vehicle, the state error is denoted as

5 $$\begin{aligned} \left\{ {\begin{array}{*{20}{l}} {{\Delta q_{i}}(t) = {q_i}(t) - ({q_0}(t)-i{q_{des}}})\\ {{\Delta v_{i}}(t) = {v_i}(t) - {v_0(t)}} \end{array}} \right. \end{aligned}$$

Define ${\zeta _i} = {{[{\Delta q_{i}}\quad {\Delta v_{i}}\quad {a_i}]}^T}$, $u_i=a_{des,i}$, the system state space equation is

6 $$\begin{aligned} \left\{ \begin{array}{l} {{{\dot{\zeta }}}_i}(t) = {\tilde{A}}_i{\zeta _i}(t) + {{\tilde{B}}_i}{u_i}(t) + {{\tilde{E}}_i}\omega (t)\\ {y_i}(t) = {\tilde{C}}_i{\zeta _i}(t) \end{array} \right. \end{aligned}$$

where

$$\begin{aligned} {\tilde{A}}_i= & {} \left[ {\begin{array}{*{20}{c}} 0&{}1&{}0\\ 0&{}0&{}{ 1}\\ 0&{}0&{}{- \frac{1}{{{\sigma _i}}}} \end{array}} \right] \;\,{{\tilde{B}}_i} = \left[ {\begin{array}{*{20}{c}} 0\\ 0\\ {\frac{1}{{{\sigma _i}}}} \end{array}} \right] \;\,{{\tilde{E}}_i} = \left[ {\begin{array}{*{20}{c}} 0\\ -1\\ 0 \end{array}} \right] \\ {{\tilde{C}}_i}= & {} \mathrm{{diag}}(\mathrm{{1}},\,\mathrm{{1}},\;\mathrm{{0}}),\;\;\;\omega (t) = {a_0}(t) \end{aligned}$$

#### Remark 2

The leader vehicle’s acceleration of ${a_0}(t)$ is a sort of “reference” for the vehicle $i \ge 1$ since the value of ${a_0}$ is already known *a priori* by vehicle to vehicle communication.

### Objective of vehicle platoon control

#### Definition 1

^27^ (Predecessor-leader following string stability): Assume that at some time instant *t*, if the desired velocity of the leader vehicle changes, the state of (5) asymptotically converges to its equilibrium, and the inter-vehicle gap error of following vehicles satisfies accordingly

7 $$\begin{aligned} {\max _{t \ge 0}}\left| {{\Delta q_{i}}(t)} \right| \le {\lambda _i}\;{\max _{t \ge 0}}\left| {{\Delta q_{i-1}}(t)} \right| \end{aligned}$$

and

8 $$\begin{aligned} {\max _{t \ge 0}}\left| {{\Delta q_{i}}(t)} \right| \le {\lambda _i}\; {\max _{t \ge 0}}\left| {{\Delta q_{1}}(t)} \right| \end{aligned}$$

**Figure 2:**
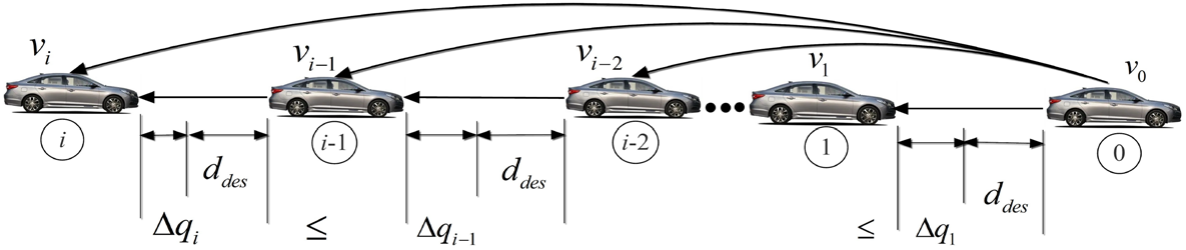
Vehicle platoons with string stability.

Note that for any vehicle $i\,$, if there exists a constant ${\lambda _i} \in (0,1)$, such that (7) and (8) are satisfied, then the vehicle platoon is string stable as shown in Fig. 2.

The objectives of the control of a platoon are summarized as follows:

The inter-vehicle gap should maintain a desired safe distance, and the velocity of the vehicles should keep the same:

9 $$\begin{aligned} \left\{ {\begin{array}{*{20}{l}} {minmize\left\| {{\Delta v_{i}}(t) - 0} \right\| _2^2 = 0,\quad \forall i \in {N_{\left[ {1,\;{} M} \right] }}}\\ {minmize\left\| {{\Delta q_{i}}(t) - 0} \right\| _2^2 = 0,\quad \forall i \in {N_{\left[ {1,\;M} \right] }}} \end{array}} \right. \end{aligned}$$

Furthermore, to guarantee that the vehicle platoon maintains steady formation driving, the following constraints should be satisfied.

1. Minimum safety distance: The distance between any front and rear vehicles should maintain a minimum safe distance to avoid collisions,
  10 $$\begin{aligned} {\Delta q_{i,\textrm{mi}}} \le {\Delta q_{i}}(t)\le {\Delta q_{i,\textrm{ma}}},\quad \forall t \ge 0 \end{aligned}$$
  where ${\Delta q_{i,\textrm{ma}}}$ and ${\Delta q_{i,\textrm{mi}}}$ are the maximum and minimum inter-vehicle gap error.
2. Consistency: The relative velocity deviation of vehicles has to be satisfied,
  11 $$\begin{aligned} {\Delta v_{i,\textrm{mi}}} \le {\Delta v_{i}}(t)\le {\Delta v_{i,\textrm{ma}}},\quad \forall t \ge 0 \end{aligned}$$
  where ${\Delta v_{i,\textrm{mi}}}$ and ${\Delta v_{i,\textrm{ma}}}$ are the minimum and maximum velocity errors.
3. Passenger comfort: During acceleration or deceleration, the control input needs to be within an admissible region:
  12 $$\begin{aligned} {u_{i,\textrm{mi}}} \le {u_i}(t) \le {u_{i,\textrm{ma}}},\quad \forall t \ge 0 \end{aligned}$$
  where $u_{i,\textrm{mi}}$ and $u_{i,\textrm{ma}}$ are the allowed minimum and maximum control input.

## Controller design

**Figure 3:**
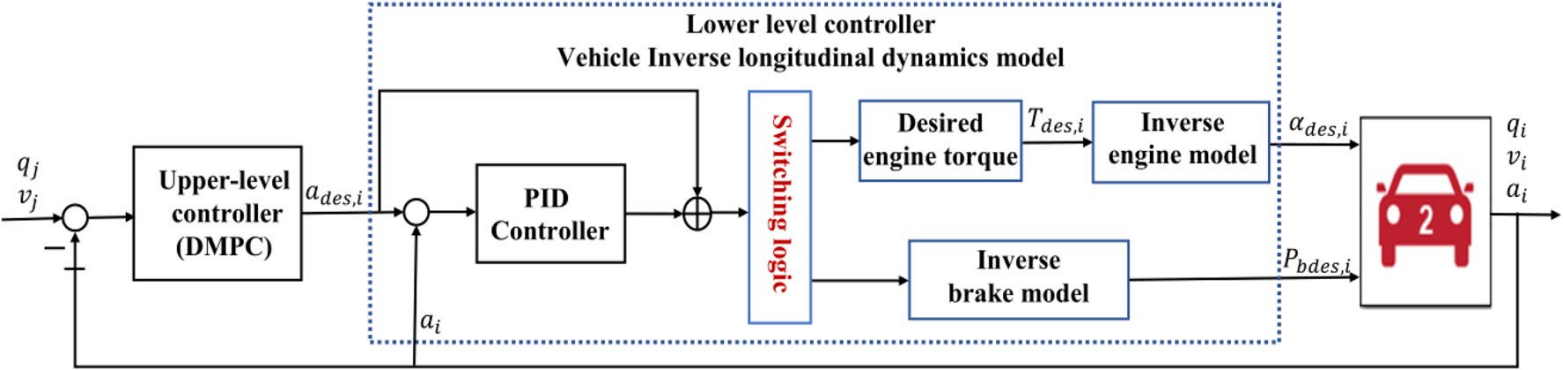
The framework of hierarchical control of the $i^{th}$ vehicle.

A hierarchical control framework is employed to achieve the vehicle platoon driving. The hierarchical control framework is illustrated in Fig. 3, where an upper-level DMPC is designed to achieve vehicle platoon control. A feedforward controller in the lower-level controller adopts feedback linearization technology to realize the adjustment of the driving and braking, and a PID controller to eliminate the influence of unmodeled dynamics and uncertainties. In Fig. 3$q_j$ is the position of adjacent vehicles; $v_j$ is the velocity of adjacent vehicles; $p_{bdes,i}$ is the desired brake pressure; $\alpha _{des,i}$ is the desired throttle angle.

### DMPC algorithm with guaranteed string stability

Denote the prediction horizon as $N_p$, sampling time $T_s > 0$. The updated time for each vehicle is denoted as

13 $$\begin{aligned} {t_\delta } = T_s \delta \end{aligned}$$

where $$\delta \in {N_{ {\left[ {0,\,\infty } \right] }}}$$.

For $p \in {N_{ {\left[ {0,\,{N_p} - 1} \right] } }}$, define three types of control inputs sequences:

- $u_i^p\left( {p;{t_\delta }} \right)$: the predicted control input sequence;
- $u_i^*\left( {p;{t_\delta }} \right)$: the optimal control input sequence;
- ${{{\hat{u}}}_i}\left( {p;{t_\delta }} \right)$: the assumed control input sequence;

Accordingly, define three types of output sequences:

- $y_i^p\left( {p;{t_\delta }} \right)$: the predicted output sequence;
- $y_i^*\left( {p;{t_\delta }} \right)$: the optimal output sequence;
- ${{{\hat{y}}}_i}\left( {p;{t_\delta }} \right)$: the assumed output sequence, which is transmitted to neighboring vehicles through communication.

At time instant ${t_\delta }$, the maximum position deviation in the prediction horizon and the maximum position deviation within one sampling instant are defined as:

14 $$\begin{aligned}{} & {} {\left| {\Delta q_{i}^*\left( {p;{t_\delta }} \right) } \right| _\infty } = \mathop {\max }\limits _{p \in {N_{ {\left[ {0,\,{N_p} - 1} \right] }}}} \left| {\Delta q_{i}^*\left( {p;{t_\delta }} \right) } \right| \end{aligned}$$

15 $$\begin{aligned}{} & {} {\left| {\Delta q_{i}^*\left( {p;{t_\delta }} \right) } \right| _{\infty ,T_s} } = \mathop {\max }\limits _{p \in {N_{ {\left[ {0,\,1} \right] }}}} \left| {\Delta q_{i}^*\left( {p;{t_\delta }} \right) } \right| \end{aligned}$$

At time instant ${t_{\delta + 1}}$, the assumed control input sequence is:

16 $$\begin{aligned} {{\hat{u}}_i}\left( {p;{t_{\delta + 1}}} \right) = \left\{ {\begin{array}{*{20}{l}} {u_i^*\left( {p+1 ;{t_\delta }} \right) ,}&{}p \in {N_{ {\left[ {0,\,{N_p} - 2} \right] }}}\\ {0,}&{}{p = {N_p} - 1 } \end{array}} \right. \end{aligned}$$

For each vehicle $i\ge 1$, the sequence of control inputs is defined at time instant $t_\delta$

$$\begin{aligned} {u_i}^p(p;{t_\delta }) = \Big \{ {u_i^p\left( {0\mid {t_\delta }} \right) ,\,u_i^p\left( {1\mid {t_\delta }} \right) , \cdots ,\,u_i^p\left( {{N_p} - 1\mid {t_\delta }} \right) \,} \Big \} \end{aligned}$$

First, a local optimization problem at time instant ${t_0}$ is designed.

#### Problem 0

17a $$\begin{aligned}&\mathop {\mathrm{{minimize}}}\limits _{u_i^p\left( {p;{t_0}} \right) } {J_i}\left( {y_i^p\left( {p;{t_0}} \right) ,u_i^p\left( {p ;{t_0}} \right) } \right) \nonumber \\&\text {subject to } \nonumber \\&{{\dot{\zeta }}_i\left( {p ;{t_0}} \right) = {\tilde{A}}_i{\zeta _i}\left( {p;{t_0}} \right) + {{\tilde{B}}_i}u_i\left( {p;{t_0}} \right) + {{\tilde{E}}_i}w\left( {p;{t_0}} \right) } \end{aligned}$$

17b $$\begin{aligned}&{y_i}(p ;{t_0}) = {\tilde{C}}_i{\zeta _i}(p ;{t_0})\end{aligned}$$

17c $$\begin{aligned}&{y_i\left( {{0};{t_0}} \right) = {y_i}\left( {{t_0}} \right) } \end{aligned}$$

17d $$ \left( {1 - {c_i}} \right) {\varepsilon _i}\big | {\Delta q_{1}^*\left( {p ;{t_0}} \right) } \big | \le \big | {\Delta q_{i}^p\left( {p ;{t_0}} \right) } \big | \le \left( {1 + {c_i}} \right) {\varepsilon _i}\big | {\Delta q_{1}^*\left( {p;{t_0}} \right) } \big |,\; i\ge 2 $$

17e $$\begin{aligned}&\big |{\Delta q_{i}^p\left( {p;{t_0}} \right) } \big | \le {\rho _i}\big | {\Delta q_{1}^*\left( {p;{t_0}} \right) } \big |,\; i\ge 2\end{aligned}$$

17f $$\begin{aligned}&{{\Delta q_{i}}\left( {p;{t_0}} \right) \in \big [ {{\Delta q_{i,\textrm{mi}}},{\Delta q_{i,\textrm{ma} }}} \big ]} \end{aligned}$$

17g $$\begin{aligned}&{{\Delta v_{i}}\left( {p;{t_0}} \right) \in \big [ {{\Delta v_{i,\textrm{mi}}},{\Delta v_{i,\textrm{ma} }}} \big ]} \end{aligned}$$

17h $$\begin{aligned}&{{u_i}\left( {p;{t_0}} \right) \in \big [ {{u_{i,\textrm{mi}}},{u_{i,\textrm{ma}}}} \big ]} \end{aligned}$$

17i $$\begin{aligned}&{y_i}\left( {{N_p};{t_0}} \right) = {[0,\;0]^T} \end{aligned}$$

where

$$\begin{aligned} {J_i}\left( {y_i^p\left( { p;{t_0}} \right) ,u_i^p\left( { p;{t_0}} \right) } \right) = \sum \limits _{p=0}^{{N_p} - 1} {\big \Vert {y_i^p\left( {p;{t_0}} \right) } \big \Vert _{{Q_i}}^2 + \big \Vert {u_i^p\left( {p;{t_0}} \right) } \big \Vert _{{R_i}}^2} \end{aligned}$$

For any ${t_\delta }>{t_0}$, a new optimization problem is constructed

#### Problem 1

18a $$\begin{aligned}&\mathop {\mathrm{{minimize}}}\limits _{u_i^p\left( {p;{t_\delta }} \right) }\; {J_i}\left( {y_i^p\left( {p;{t_\delta }} \right) ,u_i^p\left( {p;{t_\delta }} \right) ,{{{\hat{y}}}_i}\left( {p;{t_\delta }} \right) ,{{{\hat{y}}}_{i - 1}}\left( {p;{t_\delta }} \right) } \right) \nonumber \\&\text {subject to } \nonumber \\&{{\dot{\zeta }}_i}\left( {p;{t_\delta }} \right) = {\tilde{A}}_i{\zeta _i}\left( {p;{t_\delta }} \right) + {{\tilde{B}}_i}{u_i}\left( {p ;{t_\delta }} \right) + {{\tilde{E}}_i}w\left( {p;{t_\delta }} \right) \end{aligned}$$

18b $$\begin{aligned}&{y_i}(p;{t_\delta }) = {\tilde{C}}_i{\zeta _i}(p;{t_\delta }) \end{aligned}$$

18c $$\begin{aligned}&{y_i\left( {{0};{t_\delta }} \right) = {y_i}\left( {{t_\delta }} \right) } \end{aligned}$$

18d $$\begin{aligned}&{\begin{array}{*{20}{l}} {{{\left| {\Delta q_{i}^p\left( {p;{t_\delta }} \right) - {{\Delta {\hat{q}}}_{i}}\left( {p;{t_\delta }} \right) } \right| }_\infty } \le {\varpi _i}(\delta )} {\min \left\{ {{{\big | {{{\Delta {\hat{q}}}_{i - 1}}\left( {p;{t_\delta }} \right) } \big |}_{\infty ,T_s }},{{\big | {\Delta q_{i}^p\left( {p;{t_\delta }} \right) } \big |}_{\infty ,T_s },{{\big | {{{\Delta {\hat{q}}}_{1}}\left( {p;{t_\delta }} \right) } \big |}_{\infty ,T_s }}}} \right\} }\end{array}} \end{aligned}$$

18e $$\begin{aligned}&{{\Delta q_{i}}\left( {p ;{t_\delta }} \right) \in \big [ {{\Delta q_{i,\textrm{mi}}},{\Delta q_{i,\textrm{ma} }}} \big ]} \end{aligned}$$

18f $$\begin{aligned}&{{\Delta v_{i}}\left( {p;{t_\delta }} \right) \in \big [ {{\Delta v_{i,\textrm{mi} }},{\Delta v_{i,\textrm{ma} }}} \big ]} \end{aligned}$$

18g $$\begin{aligned}&{{u_i}\left( {p;{t_\delta }} \right) \in \big [ {{u_{i,\textrm{mi}}},{u_{i,\textrm{ma} }}} \big ]} \end{aligned}$$

18h $$\begin{aligned}&{y_i}\left( {{N_p};{t_\delta }} \right) = {\big [0,\;0\big ]^T} \end{aligned}$$

where

$$\begin{aligned}{} & {} {J_i}\left( {y_i^p\left( {p;{t_\delta }} \right) ,u_i^p\left( {p;{t_\delta }} \right) ,{{{\hat{y}}}_i}\left( {p;{t_\delta }} \right) ,{{{\hat{y}}}_{i - 1}}\left( {p;{t_\delta }} \right) } \right) \\{} & {} \quad = \sum \limits _{p=0}^{{N_p} - 1} {\big \Vert {y_i^p\left( {p;{t_\delta }} \right) } \big \Vert _{{Q_i}}^2 + \big \Vert {\left( {y_i^p\left( {p;{t_\delta }} \right) - {{{\hat{y}}}_i}\left( {p;{t_\delta }} \right) } \right) } \big \Vert _{{F_i}}^2} + \big \Vert {\left( {y_i^p\left( {p;{t_\delta }} \right) - {{{\hat{y}}}_{i - 1}}\left( {p;{t_\delta }} \right) } \right) } \big \Vert _{{G_i}}^2 + {\big \Vert {u_i^p\left( {p;{t_\delta }} \right) } \big \Vert ^2}_{{R_i}} + {\big \Vert {\Delta u_i^p\left( {p;{t_\delta }} \right) } \big \Vert ^2}_{{W_i}} \end{aligned}$$

and ${Q_i}$, ${F_i}$, ${G_i}$, ${R_i}$ and ${W_i}$ are weighting matrices. Note that $\Vert x_i\Vert _{P_i}^2 = {x_i^T{P_i}{x_i}}$ with ${P_i}\in {\mathbb {R}^{n\times n}}$ and ${P_i}>0$ for a vector $x_i\in {\mathbb {R}^n}$. Since the leader vehicle is uncontrolled, the term ${G_1}=0$. The term ${\Vert {( {y_i^p( {p;{t_\delta }} ) - {{{\hat{y}}}_i}( {p;{t_\delta }})} )} \Vert _{{F_i}}^2}$ is the penalty of the error of the sequence of the $i^{th}$ vehicle and its assumed output sequence; the term ${\Vert {( {y_i^p ( {p;{t_\delta }} ) - {{{\hat{y}}}_{i - 1}}( {p;{t_\delta }})} )} \Vert _{{G_i}}^2}$ is the penalty between the predicted and the assumed output sequence from the communication vehicle; the terms ${\varepsilon _i}, {c_i}, {\rho _i}\in (0,1)$ and ${\varpi _i}(\delta )$ are the parameters to be determined to ensure string stability of vehicle platoons.

Constraints (17d), (17e), together with (18d) will guarantee string stability; (17f), (17g), (18e), (18f), (17h) and (18g) are the constraints; (17i) and (18h) are the terminal equality constraint to ensure asymptotic consensus.

The distributed model predictive control scheme to ensure string stability is as Algorithm 1.
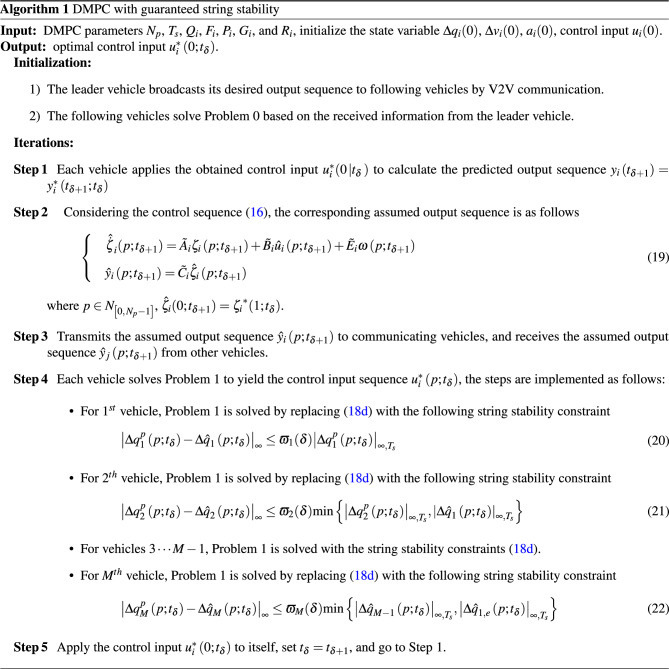


#### Remark 3

A synchronous distributed model prediction controller is presented for the vehicle platoon, where the following vehicle solves its optimization problem synchronously. Since each vehicle does not know the predicted output sequence of other vehicles, the assumed output sequences are used to replace the actual predicted output sequences in the optimization problems.

#### Remark 4

A qualitative analysis of the performance of longitudinal tracking with the proposed control scheme is performed in this paper, whereas other important issues including communication delay and packet loss, parameter uncertainty, and measurement noise of sensors will be our future research direction.

**Asymptotic consensus of DMPC**

#### Theorem 1

For Algorithm 1, if weight values of Problem 1 satisfy

23 $$\begin{aligned} {F_i} > {G_{i + 1}}, \quad i\ge 1, \end{aligned}$$

with ${G_1}=0$. Then, a vehicle platoon under Algorithm 1 is asymptotic consensus.

#### Proof

Define the sum of objective function as a candidate Lyapunov function

$$\begin{aligned} J_{\Sigma } ^*\left( {y({t_\delta })} \right) = \sum \limits _{i = 1}^M {{J_i}^*\left( {y_i^*(p;{t_\delta }),u_i^*(p;{t_\delta }),{{{\hat{y}}}_i}(p;{t_\delta }),{{{\hat{y}}}_{i - 1}}(p;{t_\delta })} \right) } \end{aligned}$$

At the time instant ${t_\delta }$, the sum of objective function is

24 $$\begin{aligned} {J_{\Sigma } ^*( {y({t_\delta })})} {{{ = }}\sum \limits _{i = 1}^M \bigg \{{ {\sum \limits _{p = 0}^{{N_p} - 1} {\big [ {\big \Vert {y_i^*( {p;{t_\delta }}} \big \Vert _{{Q_i}}^2 + } } } } } { \big \Vert {( {y_i^*( {p;{t_\delta }} ) - {{\hat{y}}_i}( {p;{t_\delta }} )} )} \big \Vert _{{F_i}}^2 { + \big \Vert {( {y_i^*( {p;{t_\delta }} ) - {{\hat{y}}_{i - 1}}( {p;{t_\delta }} )} )} \big \Vert _{{G_i}}^2}+{{\big \Vert {u_i^*( {p;{t_\delta }})} \big \Vert }^2}_{{R_i}}+ {{\big \Vert {\Delta u_i^*( {p;{t_\delta }} )} \big \Vert }^2}_{{W_i}} \big ] \bigg \} } \end{aligned}$$

Similarly, at the time instant ${t_{\delta + 1}}$, the sum of the objective function is

25 $$\begin{aligned} J_{\Sigma } ^*( {y({t_{\delta + 1}})}) = \sum \limits _{i = 1}^{M} J_i^*( {y_i^*(p;{t_{\delta + 1}}),u_i^*(p;{t_{\delta + 1}})},{{{{\hat{y}}}_i}(p;{t_{\delta + 1}}),{{{\hat{y}}}_{i - 1}}(p;{t_{\delta + 1}})}) \end{aligned}$$

At the time instant ${t_{\delta + 1}}$, since $u_i^p\left( {p;{t_{\delta + 1}}} \right) = {{{\hat{u}}}_i}\left( {p;{t_{\delta + 1}}} \right)$ is a feasible control sequence (but suboptimal) for Problem 1, the sum of objective function is bounded

26 $$\begin{aligned} J_{\Sigma }^*( {y({t_{\delta + 1}})}) \mathop \le \sum \limits _{i = 1}^M {J_i}\left( {{{{\hat{y}}}_i}(p;{t_{\delta + 1}}),{{{\hat{u}}}_i}(p;{t_{\delta + 1}})}\right. , {{{{\hat{y}}}_i}(p;{t_{\delta + 1}}),{{{\hat{y}}}_{i - 1}}(p;{t_{\delta + 1}})}) \end{aligned}$$

According to (16) and (19), one has

27 $$\begin{aligned}{} & {} {J_\Sigma ^*\left( {y({t_{\delta + 1}})} \right) }\nonumber \\{} & {} \quad { \le \sum \limits _{i = 1}^M {{J_i}} (y_i^*(p + 1;{t_\delta }),u_i^*(p + 1;{t_\delta }),y_i^*(p + 1;{t_\delta }),y_{i - 1}^*(p + 1;{t_\delta }))}\nonumber \\{} & {} \quad {{ = }}\sum \limits _{i = 1}^M \bigg \{ \sum \limits _{p = 0}^{{N_p} - 2} {\bigg [\big \Vert {y_{_i}^ * ( {p + 1;{t_\delta }} )} \big \Vert _{{Q_i}}^2+ } \big \Vert {\big ( {y_{_i}^ * ( {p+ 1;{t_\delta }} ) - y_{_i}^ * ( {p+ 1;{t_\delta }})} \big )} \big \Vert _{{F_i}}^2 + \big \Vert { \big ( {y_{_i}^ * ( {p + 1;{t_\delta }} ) - y_{_{i - 1}}^ * ( {p + 1;{t_\delta }} )}\big )} \big \Vert _{{G_i}}^2\nonumber \\{} & {} \qquad + \big \Vert u_i^*(p+1;{t_\delta }){^2}\big \Vert _{{R_i}}^2 + \big \Vert \Delta u_i^*(p+1;{t_\delta })\big \Vert _{{W_i}}^2\bigg ]\bigg \} \end{aligned}$$

In terms of (24) and (27), the following inequality is yielded

28 $$\begin{aligned}{} & {} {J_{\Sigma } ^*\left( {y({t_{\delta + 1}})} \right) - J_{\Sigma } ^*\left( {y({t_\delta })} \right) }\nonumber \\{} & {} \quad {{ = }}-\sum \limits _{i = 1}^M {\bigg [\big \Vert {y_{_i}^ * ( {0;{t_\delta }} )} \big \Vert _{{Q_i}}^2+ } \big \Vert {\big ( {y_{_i}^ * ( {0;{t_\delta }} ) - {{\hat{y}}}_{_i}( {0;{t_\delta }})} \big )} \big \Vert _{{F_i}}^2 + \big \Vert { \big ( {y_{_i}^ * ( {0;{t_\delta }} ) - {{\hat{y}}}_{_{i - 1}} ( {0;{t_\delta }} )}\big )} \big \Vert _{{G_i}}^2\nonumber \\{} & {} \qquad + \big \Vert u_i^*(0;{t_\delta }){^2}\big \Vert _{{R_i}}^2 + \big \Vert \Delta u_i^*(0;{t_\delta })\big \Vert _{{W_i}}^2\bigg ]+ \sum \limits _{p=1}^{{N_p} - 1} \Delta _{p} \end{aligned}$$

where

$$\begin{aligned} \Delta _{p} = \sum \limits _{i = 1}^M {\Big [ {\big \Vert {\big ( {y_{_i}^ * \left( {p;{t_\delta }} \right) - y_{_{i - 1}}^ * \left( {p;{t_\delta }} \right) } \big )} \big \Vert _{{G_i}}^2} \big .} - \big \Vert {\left( {y_i^ * \left( {p;{t_\delta }} \right) - {{{\hat{y}}}_{i - 1}}\left( {p;{t_\delta }} \right) } \right) } \big \Vert _{{G_i}}^2 \Big . { - \big \Vert {\left( {y_i^ * \left( {p;{t_\delta }} \right) - {{{\hat{y}}}_i}\left( {p;{t_\delta }} \right) } \right) } \big \Vert _{{F_i}}^2} \Big ] \end{aligned}$$

Due to the triangle inequality,

29 $$\begin{aligned}{} & {} \Delta _{p} \le \sum \limits _{i = 1}^M {\Big [ {\big \Vert {y_{_{i - 1}}^ * \left( {p;{t_\delta }} \right) - {{{\hat{y}}}_{i - 1}}\left( {p;{t_\delta }} \right) } \big \Vert _{{G_i}}^2} \big .} \big . { - \big \Vert {\left( {y_i^ * \left( {p;{t_\delta }} \right) - {{{\hat{y}}}_i}\left( {p;{t_\delta }} \right) } \right) } \big \Vert _{{F_i}}^2} \Big ] \end{aligned}$$

Due to $G_1 = 0$, (29) is bounded by

30 $$\begin{aligned} \Delta _{p} \le \sum \limits _{i = 1}^M {\Big [ {\big \Vert {y_{_i}^ * \left( {p ;{t_\delta }} \right) - {{{\hat{y}}}_i}\left( {p ;{t_\delta }} \right) } \big \Vert _{{G_{i + 1}}}^2} \big .} \big . { - \big \Vert {\left( {y_i^ * \left( {p ;{t_\delta }} \right) - {{{\hat{y}}}_i}\left( {p ;{t_\delta }} \right) } \right) } \big \Vert _{{F_i}}^2} \Big ] \end{aligned}$$

Since $${F_i} > {G_{i + 1}}$$,

$$\begin{aligned} J_{\Sigma } ^*\left( {y({t_{\delta + 1}})} \right) - J_{\Sigma } ^*\left( {y({t_\delta })} \right) \le 0 \end{aligned}$$

Therefore, the asymptotic consensus of Algorithm 1 is guaranteed^38^. $\square$

**String stability**

#### Remark 5

If a vehicle platoon’s communication network is exactly reliable, i.e. there is no communication delay and no data packet loss, string stability with the leader-follower (LF) communication topology is examined. Suppose there exists a velocity change for the leader vehicle, according to (6), if all vehicles are homogeneous, i.e., ${{\tilde{A}}_1} = {{\tilde{A}}_2} = \cdots = {{\tilde{A}}_M}$, ${{\tilde{B}}_1} = {{\tilde{B}}_2} = \cdots = {{\tilde{B}}_M}$, then the inter-vehicle gap error ${\Delta q_{i}}$ will not change as it propagates downstream. Otherwise, if all vehicles are heterogeneous, the inter-vehicle gap error ${\Delta q_{i}}$ might change as it propagates downstream.

#### Lemma 1

Suppose that (17d) is satisfied at the initial time instant ${t_0}$, then,

31 $$\begin{aligned} \left| {\Delta q_{i}^*\left( {p;{t_0}} \right) } \right| \le {\rho _i}\left| {\Delta q_{i-1}^*\left( {p;{t_0}} \right) } \right| , \quad p \in {N_{ {\left[ {0,\,{N_p} - 1} \right] }}} \end{aligned}$$

where

32 $$\begin{aligned} {\rho _2}= & {} \left( {1 + {c _2}} \right) {\varepsilon _2}\nonumber \\ {\rho _i}= & {} \left( {\left( {1 + {c_i}} \right) /\left( {1 - {c _{i - 1}}} \right) } \right) \left( {{\varepsilon _i}/{\varepsilon _{i - 1}}} \right) , i \in {N_{ {\left[ {{3},\,{M}} \right] }}} \end{aligned}$$

#### Proof

The position deviation of the $i -1$ and *i* vehicles is given by solving Problem 0 at the initial time instant, i.e.,

33 $$\begin{aligned} \left( {1 - {c_{i-1}}} \right) {\varepsilon _{i - 1}} \left| {\Delta q_{1}^*\left( {p;{t_0}} \right) } \right| \le \left| {\Delta q_{{i-1}}^*\left( {p;{t_0}} \right) } \right| \le \left( {1 + {c_{i-1}}} \right) {\varepsilon _{i - 1}} \left| {\Delta q_{1}^*\left( {p;{t_0}} \right) } \right| \end{aligned}$$

and

34 $$\begin{aligned} \left( {1 - {c _{i}}} \right) {\varepsilon _{i}} \left| {\Delta q_{1}^*\left( {p;{t_0}} \right) } \right| \le \left| {\Delta q_{i}^*\left( {p;{t_0}} \right) } \right| \mathrm{{ }} \le \left( {1 + {c _{i}}} \right) {\varepsilon _{i}}\left| {\Delta q_{1}^*\left( {p;{t_0}} \right) } \right| \end{aligned}$$

where $p \in {N_{ {\left[ {0,\,{N_p} - 1} \right] }}}$, then (31) holds by applying the lower bound on ${\left| {\Delta q_{i-1}^*\left( {p;{t_0}} \right) } \right| }$, and the upper bound on ${\left| {\Delta q_{i}^*\left( {p ;{t_0}} \right) } \right| }$. $\square$

#### Theorem 2

At the initial time instant, if the local optimization problem of the following vehicle has a feasible solution, and the parameters satisfy:

35 $$\begin{aligned} {\rho _i} + {\rho _i}\sum \limits _{\hbar = 1}^{\delta } {{\varpi _{i-1}}} (\hbar ) + \sum \limits _{\hbar = 1}^{\delta } {{\varpi _{i}}(\hbar )} \left( {1 + {\varpi _{i-1}}(\hbar )} \right) < 1 \end{aligned}$$

where $i \in {N_{ {\left[ {{2},\,{M}} \right] }}}$, then string stability of vehicle platoons with the predecessor-follower communication topology is guaranteed.

#### Proof

At the time instant ${t_1}$, by using the triangular inequality, the position deviation of adjacent vehicles satisfies

36 $$\begin{aligned} {\left| {\Delta q_{i}^*\left( {p;{t_1}} \right) } \right| _\infty } \le {\left| {\Delta q_{i}^*\left( {p;{t_1}} \right) - {{\Delta {\hat{q}}}_{i}}\left( {p;{t_1}} \right) } \right| _\infty } + {\left| {{{\Delta {\hat{q}}}_{i}}\left( {p;{t_1}} \right) } \right| _\infty } \end{aligned}$$

According to the string stability constraint (18d), one has

37 $$\begin{aligned} {\left| {\Delta q_{i}^*\left( {p ;{t_{1}}} \right) -{{\Delta {\hat{q}}}_{i}}\left( {p ;{t_{1}}} \right) } \right| _\infty } \le {\varpi _{i}}(1){\left| {{{\Delta {\hat{q}}}_{i-1}}\left( {p;{t_{1}}} \right) } \right| _{\infty ,T_s }} \end{aligned}$$

Then, the following inequality can be concluded

38 $$\begin{aligned} {\left| {\Delta q_{i }^*\left( {p ;{t_1}} \right) } \right| _\infty } \le {\varpi _{i}}(1){\left| {{{\Delta {\hat{q}}}_{i-1}}\left( {p;{t_1}} \right) } \right| _{\infty ,T_s }} + {\left| {{{\Delta {\hat{q}}}_{i}}\left( {p ;{t_1}} \right) } \right| _\infty } \end{aligned}$$

Similarly, by using the triangular inequality, the ${(i-1)}^{th}$ vehicle satisfies

39 $$\begin{aligned} {\left| {{{\Delta {\hat{q}}}_{i-1}}\left( {p ;{t_1}} \right) } \right| _\infty } \le {\varpi _{i-1}}(1){\left| {\Delta q_{i-1}^*\left( {p ;{t_1}} \right) } \right| _{\infty ,T_s }} + {\left| {\Delta q_{i-1}^*\left( {p ;{t_1}} \right) } \right| _\infty } \end{aligned}$$

According to the definition of the assumed trajectory, and (31), the following inequality is yielded

40 $$\begin{aligned} \left| {{{\Delta {\hat{q}}}_{i}}\left( {p;{t_1}} \right) } \right| _\infty \le {\rho _i}\left| {{{\Delta {\hat{q}}}_{i-1}}\left( {p;{t_1}} \right) } \right| _\infty \end{aligned}$$

Combining (38), (39) and (40), the position deviation of adjacent vehicles at the time instant ${t_1}$ can be obtained

41 $$\begin{aligned} {\left| {\Delta q_{i}^*\left( {p ;{t_1}} \right) } \right| _\infty } \le \left[ {\left( {1 + {\varpi _{i-1}}(1)} \right) {\varpi _{i}}(1) + {\rho _i}{\varpi _{i-1}}(1)} \right] {{{\left| {\Delta q_{i-1}^*\left( {p ;{t_1}} \right) } \right| }_{\infty ,T_s }} + {\rho _i}{{\left| {\Delta q_{i-1}^*\left( {p ;{t_1}} \right) } \right| }_\infty }} \end{aligned}$$

In terms of ${\left| {\Delta q_{i}^*\left( {p;{t_1}} \right) } \right| _{\infty ,T_s }} \le {\left| {\Delta q_{i}^*\left( {p ;{t_1}} \right) } \right| _\infty }$, (41) can be rewritten as

42 $$\begin{aligned} {\left| {\Delta q_{i}^*\left( {p;{t_1}} \right) } \right| _{\infty ,T_s }} \le \left[ {\left( {1+ {\varpi _{i-1}}(1)} \right) {\varpi _{i}}(1) + {\rho _i}{\varpi _{i-1}}(1)+{\rho _i}} \right] {{} {{\left| {\Delta q_{i-1}^*\left( {p;{t_1}} \right) } \right| }_{\infty ,T_s }}} \end{aligned}$$

In terms of (38) and (39), for each vehicle *i* at the time instant ${t_2}$,

43 $$\begin{aligned} {\left| {q_{i}^*\left( {p;{t_2}} \right) } \right| _\infty } \le {\varpi _{i}}(2)\left( {1 + {\varpi _{i-1}}(2)} \right) {\left| {\Delta q_{i-1}^*\left( {p;{t_2}} \right) } \right| _{\infty ,T_s }} + \big | {{{\Delta {\hat{q}}}_{i}}\left( {p;{t_2}} \right) } \big |_\infty \end{aligned}$$

Combining constraints (18d), (20) and (41),

44 $$\begin{aligned}{} & {} {\left| {{{\Delta {\hat{q}}}_{i}}\left( {p;{t_2}} \right) } \right| _\infty }\nonumber \\{} & {} \quad \le \left[ {\left( {1 + {\varpi _{i-1}}(1)} \right) {\varpi _{i}}(1) + {\rho _i}{\varpi _{i-1}}(1)} \right] {{{\left| {\Delta q_{i-1}^*\left( {p;{t_1}} \right) } \right| }_{\infty ,T_s }} + {\rho _i}{{\left| {{{{\hat{q}}}_{i-1}}\left( {p;{t_2}} \right) } \right| }_\infty }}\nonumber \\{} & {} \quad \le \left[ {\left( {1 + {\varpi _{i-1}}(1)} \right) {\varpi _{i}}(1) + {\rho _i}{\varpi _{i-1}}(1)} \right] {\left| {\Delta q_{i-1}^*\left( {p;{t_1}} \right) } \right| _{\infty ,T_s }} + {\rho _i}{\varpi _{i-1}}(2){\left| {\Delta q_{i-1}^*\left( {p;{t_2}} \right) } \right| _{\infty ,T_s}} {\rho _i}{\left| {\Delta q_{i-1}^*\left( {p;{t_2}} \right) } \right| _\infty } \end{aligned}$$

Similarly to (42), the position deviation of adjacent vehicles at time instant ${t_2}$ can be obtained as

45 $$\begin{aligned} {{{\left| {\Delta q_{i}^*\left( {p;{t_2}} \right) } \right| }_{\infty ,T_s } } { \le \mathop {\max }\limits _{\hbar = 1,2} {{\left| {\Delta q_{i-1}^*\left( {p ;{t_\hbar }} \right) } \right| }_{\infty ,T_s }} } {\left( {{\rho _i} + {\rho _i}\sum \limits _{\hbar = 1}^2 {{\varpi _{i-1}}} (\hbar ) + \sum \limits _{\hbar = 1}^2 {{\varpi _{i}}(\hbar )} \left( {1 + {\varpi _{i-1}}(\hbar )} \right) } \right) }} \end{aligned}$$

Base on inductive reasoning, the position deviation at the time instant ${t_\delta }$ is

46 $$\begin{aligned} {{{\left| {\Delta q_{i}^*\left( {p;{t_\delta }} \right) } \right| }_{_{\infty ,T_s}}}} { \le \mathop {\max }\limits _{\hbar = 1,2 \cdots {\delta }} {{\left| {\Delta q_{i-1}^*\left( {p;{t_\hbar }} \right) } \right| }_{\infty ,T_s}} } {\left( {{\rho _i} + {\rho _i}\sum \limits _{\hbar = 1}^{\delta } {{\varpi _{i-1}}} (\hbar ) + \sum \limits _{\hbar = 1}^{\delta } {{\varpi _{i}}(\hbar )} \left( {1 + {\varpi _{i-1}}(\hbar )} \right) } \right) } \end{aligned}$$

To guarantee string stability with the predecessor-follower communication topology, i.e.,

47 $$\begin{aligned} {\left| {q_{i }^*\left( {p;{t_{\delta }}} \right) } \right| _{\infty ,T_s }} \le {\lambda _i}\mathop {\max }\limits _{\hbar = 1,2 \cdots {\delta }} {\left| {\Delta q_{i-1}^*\left( {p ;{t_\hbar }} \right) } \right| _{\infty ,T_s }},\; {\lambda _i} \in \left( {0, 1} \right) \end{aligned}$$

the parameters can be chosen such that

48 $$\begin{aligned} {\rho _i} + {\rho _i}\sum \limits _{\hbar = 1}^{\delta } {{\varpi _{i-1}}} (\hbar ) + \sum \limits _{\hbar = 1}^{\delta } {{\varpi _{i}}(\hbar )} \left( {1 + {\varpi _{i-1}}(\hbar )} \right) < 1 \end{aligned}$$

Setting ${{\varpi _i}(\hbar )}={\varpi _i}$, ${{\varpi _i}} \in {(0,1)}$, using Taylor’s formula, (48) can be rewritten as

49 $$\begin{aligned} \frac{{{\rho _i}}}{{1 - {\varpi _{i - 1}}}} + \frac{1}{{1 - {\varpi _i}}} + \frac{1}{{1 - {\varpi _{i - 1}}{\varpi _i}}} - 2 < 1 \end{aligned}$$

That is, the string stability of vehicle platoons is guaranteed, if

50 $$\begin{aligned} \frac{{{\rho _i}}}{{1 - {\varpi _{i-1}}}} + \frac{1}{{1 - {\varpi _{i}}}} + \frac{1}{{1 - {\varpi _{i-1}}{\varpi _{i}}}} < 3 \end{aligned}$$

**Figure 4:**
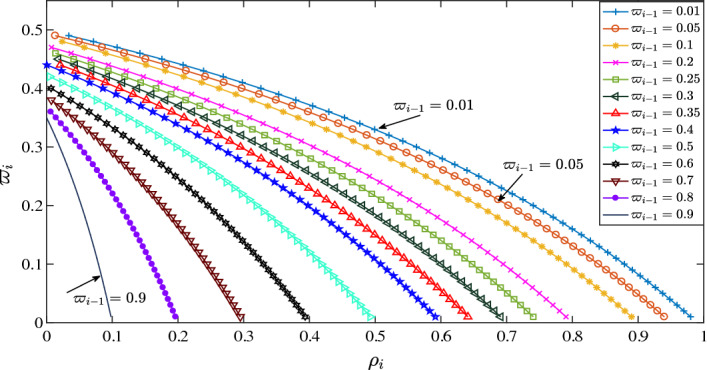
The selection of parameter values $\left\{ {{\rho _i},\,{\varpi _{i}},\,{\varpi _{i-1}}} \right\}$.

The values of $\left\{ {{\rho _i},\,{\varpi _{i}},\,{\varpi _{i-1}}} \right\}$ that satisfy (50) are shown in Fig. 4. $\square$

#### Corollary 1

At the initial time instant, if the local optimization problem of the following vehicle has a feasible solution, and the parameters satisfy:

51 $$\begin{aligned} {\rho _i} + {\rho _i}\sum \limits _{\hbar = 1}^{\delta } {{\varpi _{1}}} (\hbar ) + \sum \limits _{\hbar = 1}^{\delta } {{\varpi _{i}}(\hbar )} \left( {1 + {\varpi _{\hbar }}(\hbar )} \right) < 1 \end{aligned}$$

where $i \in {N_{ {\left[ {{2},\,{M}} \right] }}}$, then string stability of vehicle platoons with the LF communication topology is guaranteed.

Since the proof of Corollary 1 is similar to the proof of Theorem 2, it is omitted.

#### Theorem 3

Under Algorithm 1, if the local optimization problem of the following vehicle has a feasible solution at the initial time instant, and the parameters satisfy (35) and (51) simultaneously, then string stability of vehicle platoons with the PLF communication topology is guaranteed.

The proof of Theorem 3 is omitted since it can be obtained directly by using Theorem 2 and Corollary 1.

### The lower-level controller

**Figure 5:**
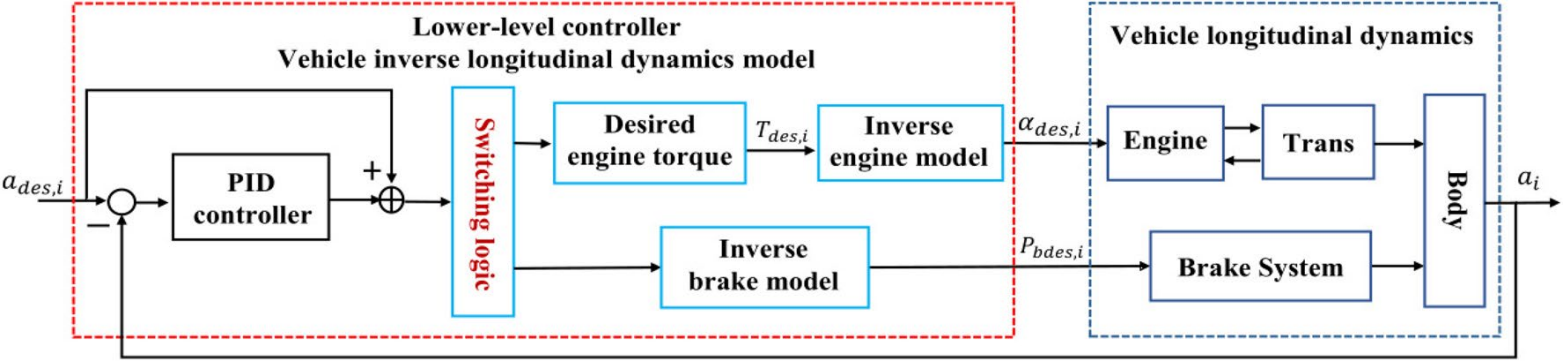
The diagram of the lower-level controller of the $i^{th}$ vehicle.

The lower-level feedforward and feedback control strategy first transforms desired acceleration into the desired throttle angle and brake pressure through an inverse longitudinal dynamics model of vehicles, and then eliminates the influence of unmodeled dynamics and uncertainties by a PID controller. The diagram of the lower-level feedforward and feedback control strategy is illustrated in Fig. 5.

*1) In the process of acceleration:* The desired acceleration is calculated^39^, i.e.,

52 $$\begin{aligned} {{m_i}{a_{des,i}} = {F_{x,i}} - \frac{1}{2}{C_{d,i}}{{{\bar{A}}_i}}{\mu }_i {{v_i}^2} - m_igf_i} \end{aligned}$$

where ${F_{x,i}}$ is the driving force of vehicles.

The engine provides a longitudinal force for the driving wheels, i.e.,

53 $$\begin{aligned} {F_{x,i}} = \frac{{{i_{g,i}}{i_{o,i}}{\eta _{T,i}}}}{{{r_{eff,i}}}}{T_{des,i}} \end{aligned}$$

where ${i_{g,i}}$ is the transmission gear ratio, and ${i_{o,i}}$ is the ratio of final gear.

Considering (52) and (53), the desired engine torque can be calculated

54 $$\begin{aligned} {{T_{des,i}} = \frac{{\left( {m_i{a_{des,i}} + \frac{1}{2}{C_{d,i}}{{\bar{A}}_i}{\mu _i} {{v_i}^2} + m_igf_i} \right) {r_{eff,i}}}}{{{i_{g,i}}{i_{o,i}}{\eta _{T,i}}}}} \end{aligned}$$

**Figure 6:**
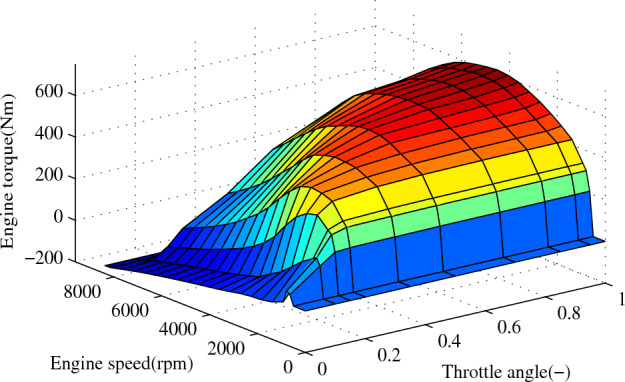
Engine torque map of the ith vehicle.

According to the engine torque ${T_{des,i}}$, the engine torque characteristic map of the F-Class vehicle from CarSim software shown in Fig. 6, and the engine speed ${w_{e,i}}$, the desired throttle angle can be obtained by the inverse look-up table method^10^, i.e.,

55 $$\begin{aligned} {{\alpha _{des,i}} = f_i^{-1}\left( {{T_{des,i}},{w_{e,i}}} \right) } \end{aligned}$$

and the term $f_i^{-1} :( {{T_{des,i}}} ) \times ( {{w_{e,i}}} ) \rightarrow ( {{\alpha _{des,i}}} )$ represents a mapping of the $i^{th}$ vehicle.

*2) In the process of braking:* The vehicle dynamics in the process of braking is as follows^39^:

56 $$\begin{aligned} {m_i{a_{{{des,i}}}} =-{F_{b,i}} -\frac{1}{2}{C_{d,i}}{{\bar{A}}_i}{\mu }_i {{v_i}^2} - m_igf_i} \end{aligned}$$

where ${F_{b,i}}$ represents the braking force of the vehicle. The desired braking force satisfy^40^,

57 $$\begin{aligned} {{F_{b,i}}= {K_{s,i}}{p_{bdes,i}}} \end{aligned}$$

where ${K_{s,i}}$ is the braking coefficient, and

58 $$\begin{aligned} {{K_{s,i}}= \frac{{{T_{bf,i}} - {T_{br,i}}}}{{p_{b,i}}{{r_{eff,i}}}}} \end{aligned}$$

the term ${p_{b,i}}$ is the braking pressure, ${T_{bf,i}}$ and ${T_{br,i}}$ are the braking torques of the front and rear wheels, respectively.

According to (57), the relationship between braking pressure and acceleration is

59 $$\begin{aligned} {{p_{b\mathrm{{des,i}}}} = \frac{{\left| { - m_i{a_{des,i}} -\frac{1}{2}{C_{d_i}}{\bar{A}}_i{\mu }_i {{v_i}^2} - m_igf_i} \right| }}{{{K_{s,i}}}}} \end{aligned}$$

After obtaining the current desired throttle angle and braking pressure, a PID controller is used to correct the error, i.e.,

60 $$\begin{aligned} {{\alpha _i} = {a_{{{des,i}}}} + {K_{P}}\left( {{a_{{{des,i}}}} - {a_i}} \right) + {K_{I}}\int {\left( {{a_{{{des,i}}}} - {a_i}} \right) } dt} {+ {K_{D}}\frac{{d\left( {a_{{{des,i}}} - {a_i}} \right) }}{{dt}}} \end{aligned}$$

where ${K_{P}}$, ${K_{I}}$, and ${K_{D}}$ are parameters of the PID controller.

*3) Throttle-brake switching logic:* To improve fuel economy and passenger comfort, and to avoid the frequent switching of drive and brake, a threshold-based throttle switching strategy is implemented in this paper^10^. First, the vehicle velocity $v_{i,{(0)}}$ and maximum acceleration $a_{i,{(0)}}$ without throttle angle and brake pressure are calibrated, which is shown in Table 1.

**Table 1:** Velocity-acceleration table without throttle angle and brake pressure of the $i^{th}$ vehicle.

Velocity(*km*/*h*)	0	10	20	30	40
Acceleration($m/s^{2}$)	0	−0.0021	−0.0083	−0.0186	−0.0331
Velocity(*km*/*h*)	50	60	70	80	90
Acceleration($m/s^{2}$)	−0.0518	−0.0745	−0.1014	−0.1428	−0.1915
Velocity(*km*/*h*)	100	110	120	130	140
Acceleration($m/s^{2}$)	−0.2449	−0.2994	−0.3542	−0.4130	−0.4756

**Figure 7:**
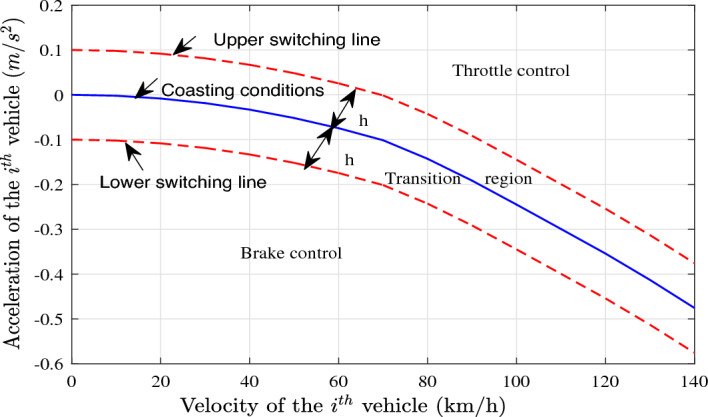
Coasting simulation curve of the ith vehicle.

A throttle-brake switching logic is designed according to Table 1, which is shown in Fig. 7 as well. Set the transition belt with the width of 2*h*, where $h = 0.1$^41^.

(i) When the desired acceleration ${a_{des,i}}$ is above upper switching line, i.e., ${a_{des,i}} \ge {a_{i{(0)}}} + h$, the throttle control is triggered;
(ii) When the desired acceleration ${a_{des,i}}$ is below lower switching line, i.e., ${a_{des,i}} \le {a_{i,{(0)}}} - h$, the brake control is launched;
(iii) When the desired acceleration ${a_{des,i}}$ is inside the transition belt, i.e., ${a_{i,{(0)}}} - h \le {a_{des,i}} \le {a_{i,{(0)}}} + h$, neither throttle control nor brake control is carried out.

#### Remark 6

The vehicle driving equation (52) and the brake equation (56) are consistent according to (1).

## Simulation and result analysis

**Figure 8:**
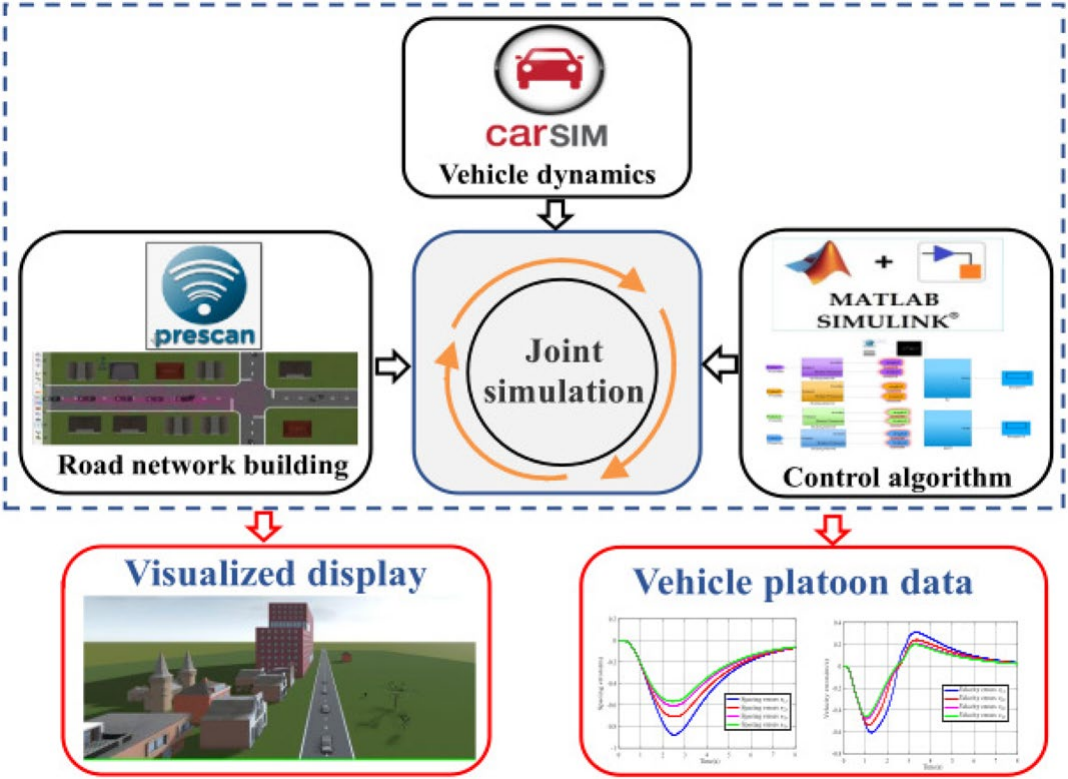
The framework of the joint simulation platform.

A vehicle platoon consists of five vehicles, i.e., one leader vehicle, and four following vehicles. A joint simulation platform with PreScan, CarSim, and Simulink is constructed shown in Fig. 8, where Prescan provides the road environment information, CarSim provides the vehicle dynamics, and Simulink is employed to design and implement of the controller. All vehicle parameters in the joint simulation are the same except for the vehicle mass $${m _i} $$, i.e., $${{C_{d,i}}}={C_{d}}$$, $$\mu _i=\mu $$, $${{\bar{A}}_i}={\bar{A}}$$, $${{\sigma }_i} = {\sigma } $$, $${\eta _{T,i}} = {\eta _{T}}$$, $${r_{eff,i}}={r_{eff}}$$, $$f_{i}=f$$, $${i_{o,i}}={i_{o}}$$, $${i_{g,i}}={i_{g}}$$. In the joint simulation, the vehicle employs an eight-speed automatic transmission. Set $${m _0}=1820\,\textrm{kg}$$, $${m _1}=1984\,\textrm{kg}$$, $${m_2}=1942\,\textrm{kg}$$, $${m _3}=1898\,\textrm{kg}$$, $${m _4}=1865\,\textrm{kg}$$. The parameter values of the F-Class vehicle from CarSim software are defined in Table 2, and the parameter values of the controller are provided in Table 3. The sampling time is chosen as $$T_s=0.2s$$, and the prediction horizon is set as $$N_p=6$$. In addition, choose the parameters of $$c_i =\varpi _i^{}$$, $${\varepsilon _2}\mathrm{{ = }}{\rho _2}/\left( {1 + {c_2}} \right) $$, $${\varepsilon _i} = ({\rho _i}*{\varepsilon _{i - 1}})/\left( {\left( {1 + {c_i}} \right) /\left( {1 - {c_{i - 1}}} \right) } \right) , i\in {N_{ {\left[ {{3},\,{M}} \right] }}}$$.

**Table 2:** The parameter values of the F-Class vehicle from CarSim software.

Parameters	Value	Parameters	Value
*g*	$9.81(\mathrm {m/s}^{2})$	$\mu$	$1.21(\mathrm {kg/m}^{3})$
${\bar{A}}$	$3(\textrm{m}^{2})$	$r_{{eff}}$	$0.353(\textrm{m})$
$\sigma$	0.5	*f*	0.01
$C_{d}$	0.3	${i_o}$	2.65
${\eta _{T}}$	0.99	${i_g}$	[4.595, 2.724, 1.864, 1.464, 1.231, 1.0, 0.824, 0.685]

**Table 3:** The parameter values of the controller.

Parameters	Value
DMPC	${Q_i}$=diag(50,20), ${F_i}$=diag(50,20), ${R_i}$=1,
	${G_i}$=diag(25,10), ${W_i}$=0.5, ${N_p}$ = 6, $T_s$= 0.2,
Constraints	$\Delta q_{i,\textrm{mi}}=-2$, $\Delta q_{i,\textrm{ma}}=2$,
	$\Delta v_{i,\textrm{mi} }=-2$, $\Delta v_{i,\textrm{ma} }=2$,
	$u_{i,\textrm{mi}}=-4$, $u_{i,\textrm{ma}}=4$,
PID	${K_P}$=0.6, ${K_I}$=0.02, ${K_D}$=0.01,
String stability	${\varpi _1}$=0.2, ${\varpi _2}$=0.3, ${\varpi _3}$=0.4, ${\varpi _4}$=0.44,
	${\rho _2}$=0.4, ${\rho _3}$=0.1, ${\rho _4}$=0.0004.

In the joint simulation, a platoon with five vehicles is interconnected by the LF communication topology and PLF communication topology, respectively. A constant distance strategy is employed, i.e., ${q_{des}} = 15\textrm{m}$. The leader vehicle in the platoon is running along a given straight road. Set the initial feasible state of the vehicles as $[{\Delta q_{i}}\;{\Delta v_{i}}] = [0\;\,0]$, $i=1,2,3,4$, respectively. When the leader vehicle accelerates, set the initial state of the leader vehicle as ${q_0}(t) = 100\textrm{m}$, ${v_0}(t) = 15\mathrm {m/s}$ and the desired velocity trajectory is given by

$$\begin{aligned} {v_0}(t) = \left\{ \begin{array}{l} 15 + 2t\;\mathrm {m/s}\quad t \le 2.5s\\ 20\;\mathrm {m/s}\;\;\;\;\;\,\;\;\;\quad t > 2.5s \end{array} \right. \end{aligned}$$

When the leader vehicle decelerates, set the initial state of the leader vehicle as ${q_0}(t) = 100\textrm{m}$, ${v_0}(t) = 20\mathrm {m/s}$ and the desired velocity trajectory is given by

$$\begin{aligned} {v_0}(t) = \left\{ \begin{array}{l} 20- 2t\;\mathrm {m/s}\quad t \le 2.5s\\ 15\;\mathrm {m/s}\;\;\;\;\;\,\;\;\;\quad t > 2.5s \end{array} \right. \end{aligned}$$

The proposed DMPC algorithm with string stability constraints is implemented in Matlab. The joint simulation performance with the LF communication topology is shown in Figs. 9, 10, 11, 12, 13 and 14. For the PLF communication topology, the acceleration performance is shown in Figs. 15, 16, 17, 18 and 19, and deceleration performance is shown in Figs. 20, 21, 22, 23 and24.

**Figure 9:**
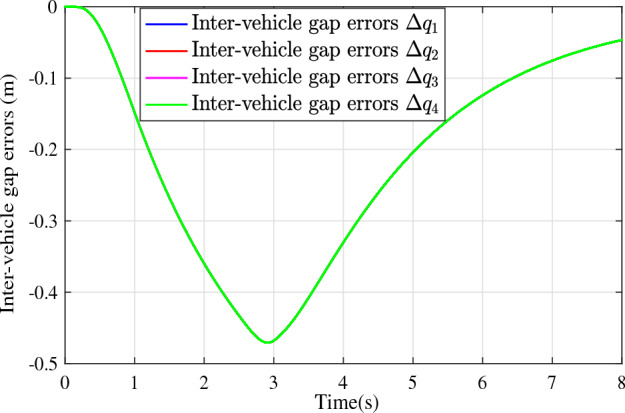
Inter-vehicle gap errors for homogeneous vehicles under LF topology.

**Figure 10:**
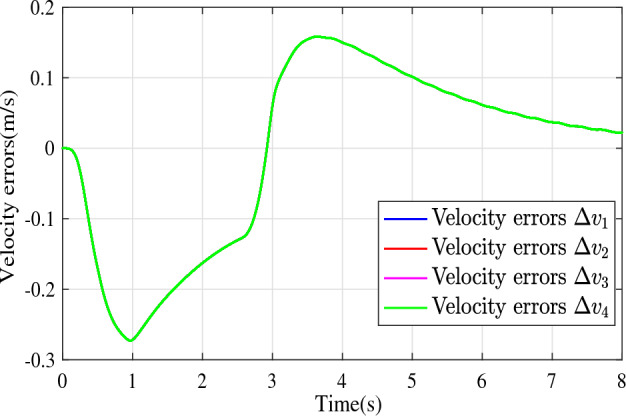
Velocity errors for homogeneous vehicles under LF topology.

**Figure 11:**
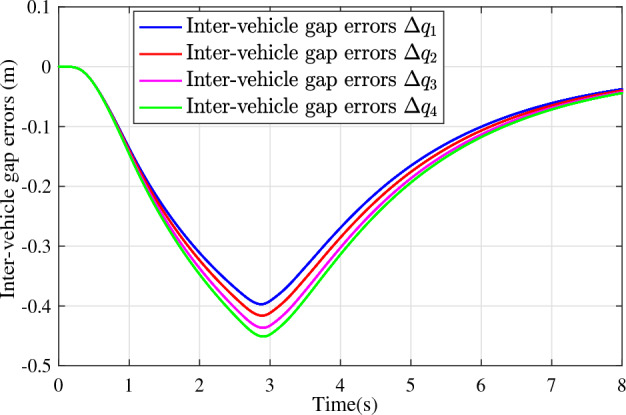
Inter-vehicle gap errors for heterogeneous vehicles under LF topology.

**Figure 12:**
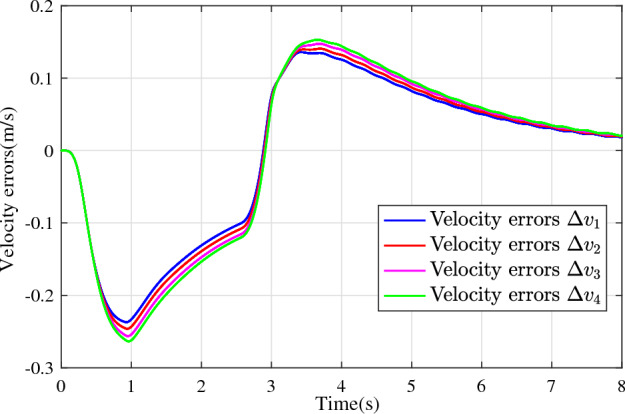
Velocity errors for heterogeneous vehicles under LF topology.

**Figure 13:**
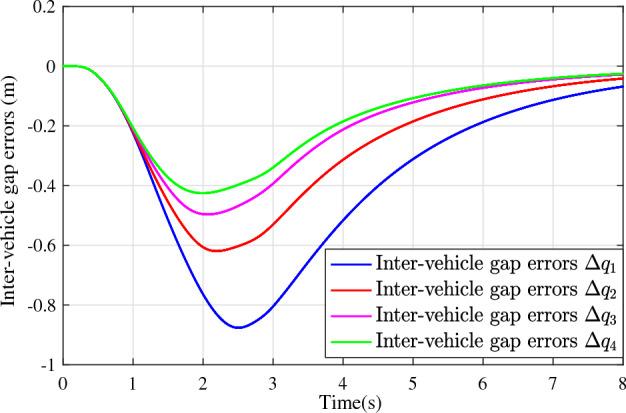
Inter-vehicle gap errors with string stability constraints under LF topology.

**Figure 14:**
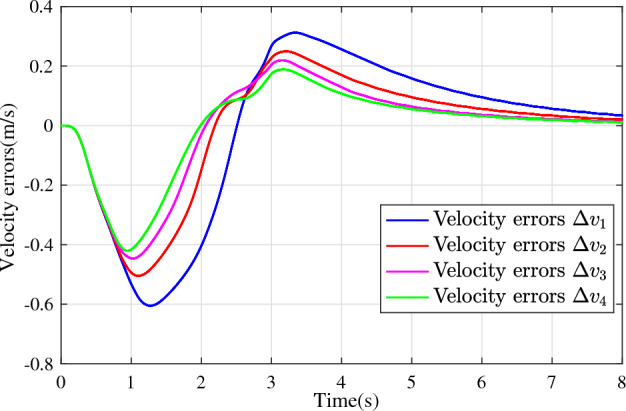
Velocity errors with string stability constraints under LF topology.

For the LF communication topology, Figs. 9-10 show the inter-vehicle gap errors and velocity errors of homogeneous vehicle platoons. Figs. 11-12 show the inter-vehicle gap errors and velocity errors of heterogeneous vehicle platoons. It can be found that when the leader vehicle’s velocity changes, if all vehicles in the platoon are homogeneous, the inter-vehicle gap errors will not be amplified as it propagates downstream; while if all vehicles are heterogeneous, the inter-vehicle gap error will be amplified as it propagates downstream. Figs. 13-14 show that the inter-vehicle gap errors and velocity errors are gradually attenuated as they propagate downstream by adopting the proposed algorithm for the heterogeneous vehicle platoon.

**Figure 15:**
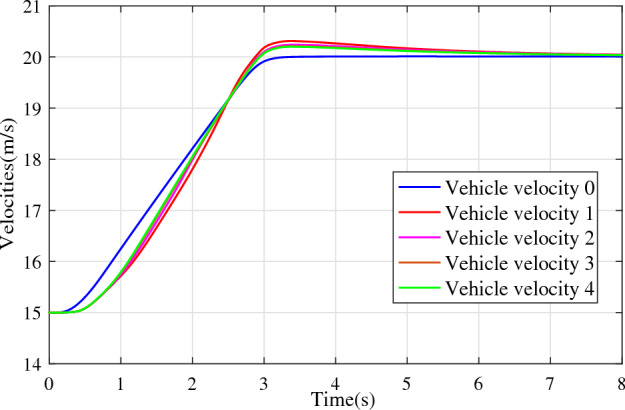
Vehicle velocities with string stability constraints under PLF topology (accelerates case).

**Figure 16:**
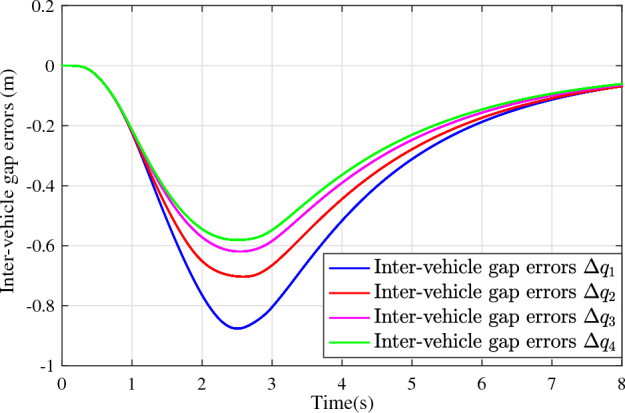
Inter-vehicle gap errors with string stability constraints under PLF topology (accelerates case).

**Figure 17:**
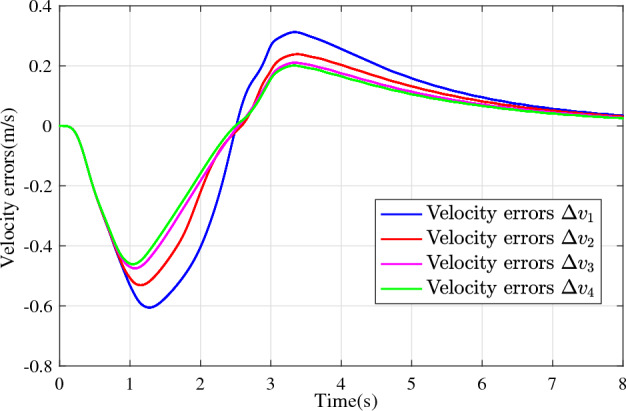
Velocity errors with string stability constraints under PLF topology (accelerates case).

**Figure 18:**
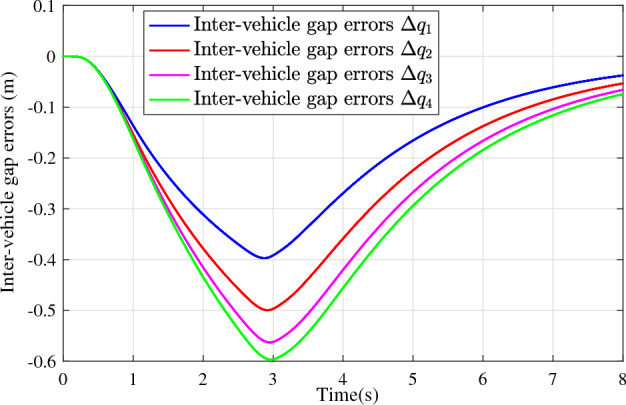
Inter-vehicle gap errors without string stability constraints under PLF topology (accelerates case).

**Figure 19:**
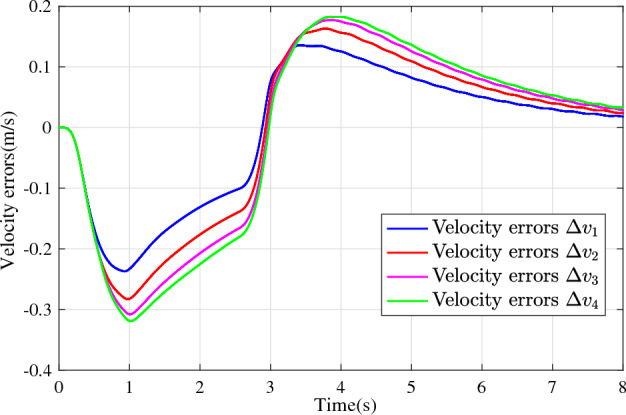
Velocity errors without string stability constraints under PLF topology (accelerates case).

For the PLF communication topology, Fig. 15 shows that the leader vehicle accelerates, and the following vehicles can track the leader vehicle and maintain consistency with the velocity of the leader vehicle. Figs. 16-17 show the inter-vehicle gap errors and velocity errors of vehicles platoons. It can be found that the inter-vehicle gap error is attenuated as it propagates downstream with the proposed DMPC algorithm. As a comparison, a DMPC without string stability constraints, and with the same controller parameters is implemented, and the results of the joint simulation are shown in Figs. 18-19. It can be seen that the inter-vehicle gap error is amplified as it propagates downstream.

**Figure 20:**
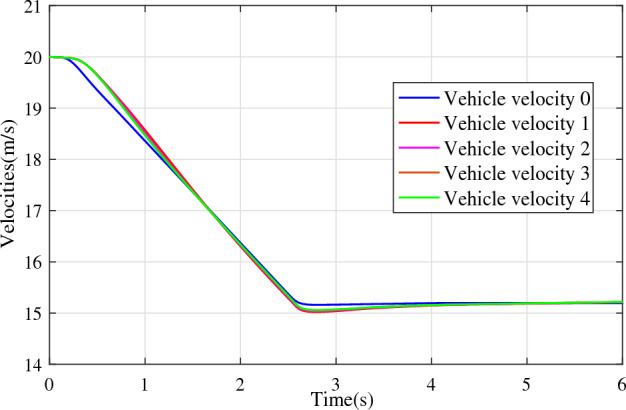
Vehicle velocities with string stability constraints under PLF topology (decelerates case).

**Figure 21:**
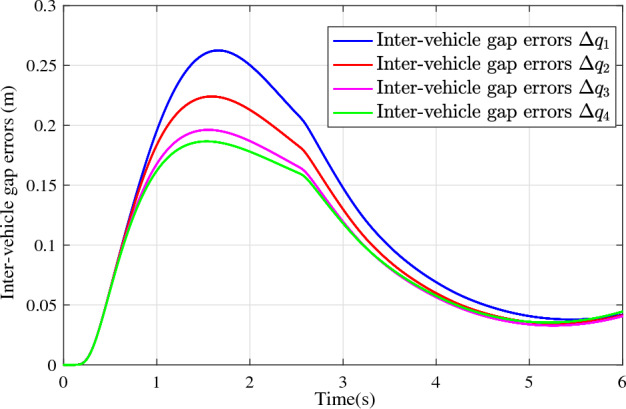
Inter-vehicle gap errors with string stability constraints under PLF topology (decelerates case).

**Figure 22:**
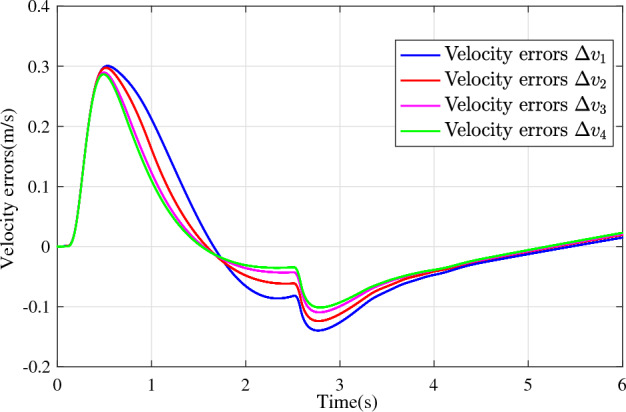
Velocity errors with string stability constraints under PLF topology (decelerates case).

**Figure 23:**
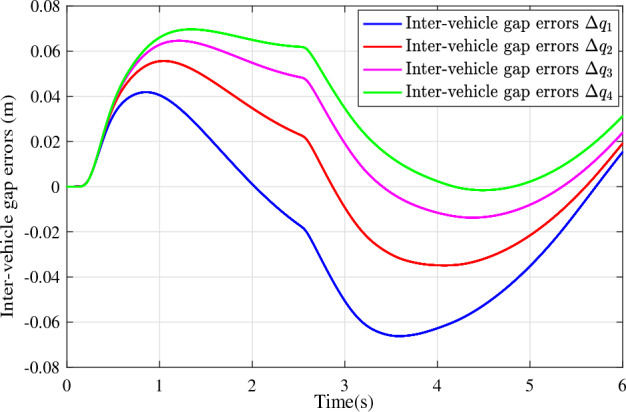
Inter-vehicle gap errors without string stability constraints under PLF topology (decelerates case).

**Figure 24:**
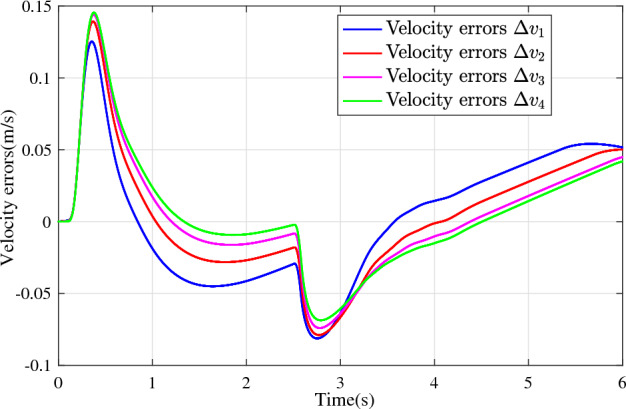
Velocity errors without string stability constraints under PLF topology (decelerates case).

Figs. 20, 21, 22, 23 and 24 show the joint simulation results when the leader vehicle decelerates. Figs. 20 shows that when the leader vehicle decelerates, the following vehicle can quickly track and keep the consistent velocity with the leader vehicle. Figs. 21-22 show that when the leader vehicle decelerates, the inter-vehicle gap error is attenuated as it propagates downstream. From the joint simulation results in Figs. 23-24, it can be found that the inter-vehicle gap error of vehicle platoons adopting the DMPC algorithm without string stability constraint is increasing.

### Remark 7

Note that the performance of the proposed control scheme should be assessed by high-fidelity tests^42^. However, the current experimental conditions of the Hardware-in-the-loop or small-scale vehicle are not yet available, and we will consider the experiment with Hardware-in-the-loop or small-scale vehicles in the future.

## Conclusion

In this paper, a hierarchical control structure was designed for communication vehicles in the platoon. Firstly, a synchronous DMPC algorithm was proposed as the upper-level controller, in which each vehicle in the platoon solves its local optimization problem synchronously to obtain the control sequence, and then transmits its assumed output sequence to neighbouring vehicles. By introducing the assumed output sequence instead of the actual predicted output sequence, the computational efficiency is improved. By adding string stability constraints and terminal equality constraints in the local optimization problem, thereby both the asymptotic consensus and string stability of vehicle platoons are guaranteed. Additionally, the sufficient condition that guarantees asymptotic consensus and string stability of vehicle platoons were given, respectively. Then, a lower-level controller was designed, where the desired control input determined by the upper-level DMPC was first transformed into the desired throttle angle and brake pressure through an inverse longitudinal dynamics model of vehicles. A PID feedback controller was employed to eliminate the influence of unmodeled dynamics and uncertainties so as to achieve the desired control performance. Finally, performance was verified by a joint simulation platform based on PreScan, CarSim and Simulink.

## Data Availability

Due to space limitation, this paper only shows partial results. The datasets generated during and/or analysed during the current study are available from the corresponding author on reasonable request.
